# Degradation of a novel magnesium alloy-based bioresorbable coronary scaffold in a swine coronary artery model

**DOI:** 10.1007/s12928-024-01023-3

**Published:** 2024-07-22

**Authors:** Sho Torii, Akiko Yamamoto, Ayako Yoshikawa, Linhai Lu, Makoto Sasaki, Shoko Obuchi, Akira Wada, Hideo Tsukamoto, Gaku Nakazawa

**Affiliations:** 1 Department of Cardiology, School of Medicine, Faculty of Medicine, Tokai University, 143 Shimokasuya, Kanagawa, 2591193 Japan; 2 Research Center for Macromolecules and Biomaterials, National Institute for Materials Science, Ibaraki, Japan; 3 Shanghai Kepan Investment and Management CO., LTD, Shanghai, China; 4 Faculty of Advanced Science and Technology, Kumamoto University, Kumamoto, Japan; 5 Japan Medical Device Technology Co., Ltd, Kumamoto, Japan; 6 Department of Cardiology, Faculty of Medicine, Kindai University, Osaka, Japan

**Keywords:** Percutaneous coronary intervention, Bioabsorbable magnesium scaffolds, Preclinical study, Elemental mapping, Controlled degradation profile

## Abstract

The objective of the study is to investigate the safety, feasibility, and degradation profile of a novel Mg alloy-based bioresorbable coronary scaffold (JFK-PRODUCT BRS) with thin struts (110 μm). Polymer- or Mg alloy-based BRSs have not replaced nondegradable metal stents because of the higher prevalence of scaffold thrombosis and restenosis in clinical practice; these poor clinical outcomes were due to inadequate scaffold designs, including thick struts (more than 150 μm) and their inappropriate degradation processes. Fourteen healthy pigs received 17 JFK-PRODUCT BRSs in the coronary arteries and were sacrificed at 1, 6, 12, 18, and 26 months after implantation. Angiography, optical coherence tomography, microfocus X-ray computed tomography (µCT), scanning electron microscopy with energy-dispersive X-ray spectrometry (SEM–EDX), and histopathological evaluation were performed. The JFK-PRODUCT had a median percent late recoil of 11.28% at 1 month. The µCT observation confirmed that scaffold discontinuity reached 64.8% at 12 months with increased scaffold inner area thereafter, suggesting artery positive remodeling. The inflammation was mild, peaked at 18 months, and decreased thereafter. The SEM–EDX analysis demonstrated gradual degradation of the scaffold with formation of inorganic deposits, presumed to be calcium phosphates. It also revealed the disappearance of calcium phosphates at 26 months, achieving almost complete replacement of the scaffold by biocomponents. The current study demonstrated the safety and feasibility of JFK-PRODUCT with a lower acute recoil rate despite its thin struts. The scaffolds were almost completely disappeared at 26 months after implantation.

**Graphical abstract:**

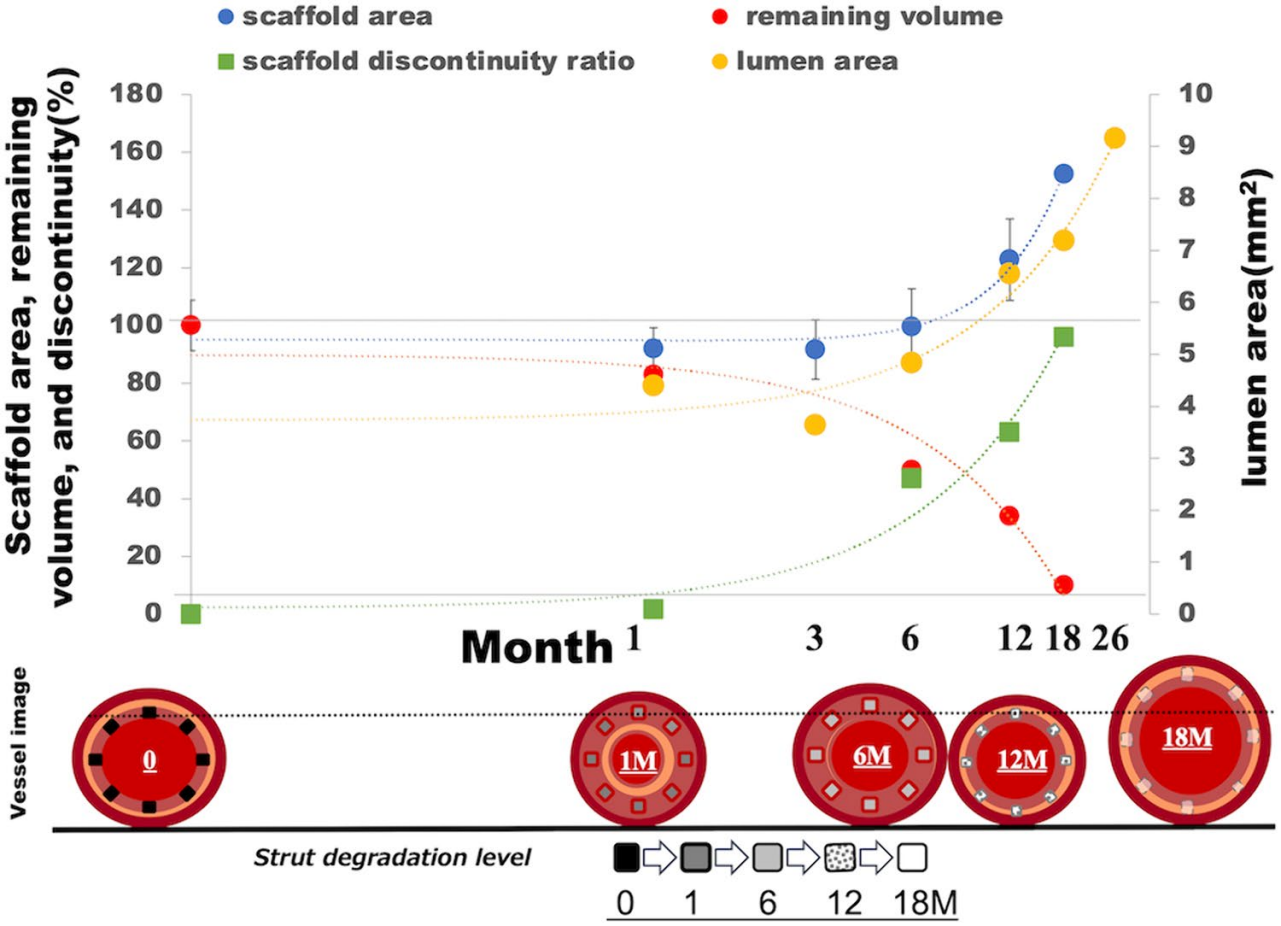

**Supplementary Information:**

The online version contains supplementary material available at 10.1007/s12928-024-01023-3.

## Introduction

Bioresorbable scaffolds (BRSs) have been introduced to counter the well-known limitations of current drug-eluting stents (DESs) in percutaneous coronary artery intervention, which include delayed healing and neoatherosclerosis, leading to restenosis and very late stent thrombosis [1, 2]. As the BRS disappears over time, it clears the above-mentioned issues of the current DESs and provides benefits to leave behind only the native vessel for future treatment strategies such as stent/scaffold implantation and coronary artery bypass graft. These expectations are based on several preclinical and clinical studies for BRSs which was completely associated with late vessel lumen enlargement and restoration of vasomotion [2–4]. Thus, BRSs have the potential to become an important treatment strategy for patients who require coronary revascularization. However, they have not yet replaced DESs because of their various imperfections.

The first BRS commercialized was poly(L-lactide)-based Absorb GT-1 (Abbott Vascular, Santa Clara, CA, USA); however, it had a significantly higher incidence of scaffold thrombosis than the Xience DES (Abbott Vascular) in a meta-analysis [5], owing to its thicker strut (157 μm) and extended degradation period (3–5 years) [3, 6]. A Mg alloy-based BRS, Magmaris (DREAMS 2G, Biotronik, Berlin, Germany), was developed and commercialized in Europe [7–9], although this also has a thick strut (150 μm) [7] to maintain a certain level of radial force. The degradation period of Magmaris is shorter than 12 months; however, the late lumen loss was numerically higher than that of the currently available DESs, mainly because of the reduced radial force in the early phase after implantation. In addition, the scaffold leaves amorphous calcium phosphates (substituting Mg alloy backbone) in the artery tissue for more than 2 years [10]. Recently, the DREAMS 3G (Biotronik, Berlin, Germany), the third generation of the Magmaris, was developed, which has a thinner strut made of a new Mg–Al alloy [11]. However, a preclinical study demonstrated that the scaffold was fully substituted with calcium phosphate within 12 months, which is similar to that of the earlier generation Magmaris [12].

Against this background, a novel BRS (JFK-PRODUCT, Japan Medical Device Technology Co. Ltd., Kumamoto, Japan) was recently developed to overcome the drawbacks of the currently available BRSs. The aim of this study was to evaluate the safety, feasibility, and degradation profile of the JFK-PRODUCT using a porcine coronary artery model for up to 26 months.

## Methods

### Test device

The Mg alloy employed in the JFK-PRODUCT was free of rare earth (RE) elements such as yttrium (Y), dysprosium (Dy), neodium (Nd), and gadolinium (Gd). The alloy surface was passivated by hydrofluoric acid and then coated with three layers: Parylene C, poly (DL-lactide-co-ε-caprolactone), and poly (DL-lactide) [13, 14]. The concentration of Sirolimus was 1.0 µg/mm^2^. The JFK-PRODUCT consisted of an open cell with a six-crown two-link scaffold with a diameter of 2.5–3 mm and a strut thickness of 110 μm [15]. (Supplemental Figure S1).

## Experimental model

The study protocol was reviewed and approved by the Institutional Animal Care and Use Committee of AccelLAB (Quebec, Canada) and Gateway Medical Innovation Center (Shanghai, China). All the animals were received aspirin (100 mg) and clopidogrel (75 mg), at least 72 h prior to intervention and continuing daily until euthanasia. The medications were crushed and mixed with their food or with an appropriate carrier. A total of 14 swine received JFK-PRODUCT in 17 coronary arteries and were sacrificed at 28 days [1 month (M), *n* = 3], 180 days (6 M, *n* = 4), 365 days (12 M, *n* = 4), 540 days (18 M, *n* = 4), and 780 days (26 M, *n* = 2) after scaffold implantation (Supplemental Table S1). After pretreatment angiography, the scaffold was deployed to achieve a balloon-to-artery ratio from 1.05 to 1.10:1 based on Quantitative coronary angiography (QCA) measurements. Detailed procedures are provided in the supplemental material.

## Quantitative coronary angiography analysis

QCA was performed using the Medis QAngio® XA 7.3 system (Medias, Leiden, the Netherlands) as previously reported. For angiograms recorded at orthogonal angles in the coronary arteries, the mean values of parameters measured or calculated from the two angles were reported. The detailed method for QCA analysis is described in the supplemental material.

## Optical coherence tomography analysis

Optical coherence tomography imaging was performed using the ILUMIEN or ILUMIEN Optics imaging system (Abbott Vascular, Santa Clara, CA, USA). After scaffold implantation, OCT analysis was performed for all treated vessels. The vessel patency, scaffold and strut adherence to the vessel wall, and constructive change of the scaffold struts were evaluated on the basis of the OCT images of implanted vessel sections. OCT was also performed before euthanasia. The detailed method for OCT analysis is described in the supplemental material.

## Scaffold harvest

The scaffolds were harvested at designated time points. After nitroglycerin was delivered to the treated arteries, angiography and OCT were performed. The hearts were harvested and perfused with lactated Ringer’s solution, and fixation perfusion was performed using neutral buffered formalin (NBF). Treated vessels were exposed to NBF for approximately 24 ± 4 h before further processing.

## Microfocus X-ray computed tomography (µCT) analysis

Before histological processing, the degradation of the scaffold and strut discontinuity were analyzed based on µCT observation (SMX-90CT, Shimadzu, Kyoto, Japan, or XT H225ST, Nikon, Tokyo, Japan) under optimal conditions for each µCT system. The remaining volume of the scaffold was estimated on the basis of histogram data using a peak fitting program [16]. Strut discontinuity was manually analyzed on the 3D images visualized using image analysis software (VGStudio MAX3.0, Volume Graphics, Heidelberg, Germany). The scaffold inner area was measured on 2D sectional images prepared at approximately 3-mm intervals from the proximal end of the scaffold. A detailed method for µCT analysis is shown in the supplemental material.

## Histological preparation and assessment

For light microscopy, the treated vessel segments were dehydrated in a graded series of ethanol and embedded in Spur’s epoxy resin. After polymerization, the proximal, middle, and distal segments, approximately 2 to 3 mm, were sawed from the devices as previously described [17–19]. Sections from the implants were sliced by a rotary microtome at 4 to 6 µm, mounted, and stained with hematoxylin and eosin (H&E), Elastin Trichrome, and modified Movat Pentachrome stains.

Morphometric analysis and histological assessment of scaffolded sections were performed as previously described [17, 20, 21]. The detailed method for morphometric analysis is described in the supplemental material. Histological assessments, such as vessel injury and inflammation score, were semi-quantitatively scored for each section. Struts with fibrin, > 10 inflammatory cells, giant cells, and calcification were counted and expressed as percentages as previously described [17, 20, 21].

## Scanning electron microscopy with energy-dispersive X-ray spectrometry (SEM–EDX) analysis

Observation of the cross-sectional morphologies and element analysis were performed by field emission-scanning electron microscopy (FE-SEM; JSM-7100F JEOL, Tokyo, Japan) equipped with energy-dispersive X-ray spectroscopy (EDX; JED-2300F, JEOL). The samples were sputter-coated with pure gold (SC-701 AT, Sanyu Electron, Tokyo, Japan) and observed at 15 kV. Additionally, elemental mapping analysis using EDX was performed to characterize the degradation layer on the implanted scaffold. EDX mapping of six elements (C, Mg, Ca, P, F, Cl) was performed at an accelerating voltage of 15 keV with a 120 μm aperture size at 200-fold magnification.

## Statistical analysis

Continuous variables with a normal distribution are expressed as the mean ± SD, while variables with a non-normal distribution are expressed as medians (interquartile range [IQR]). Categorical variables were expressed as counts and percentages, and the chi-square test or Fisher’s exact test was used for comparison. The Kruskal–Wallis test was employed to examine the differences among six independent timepoints due to the non-normal distribution of the data. A p value < 0.05 was considered statistically significant. SAS system Release 9.2 (SAS Institute Inc.) and JMP software (version 13.0, Cary, NC) were used for statistical analyses.

## Results

### Quantitative coronary angiography and optical coherence tomography analysis

**Fig. 1:**
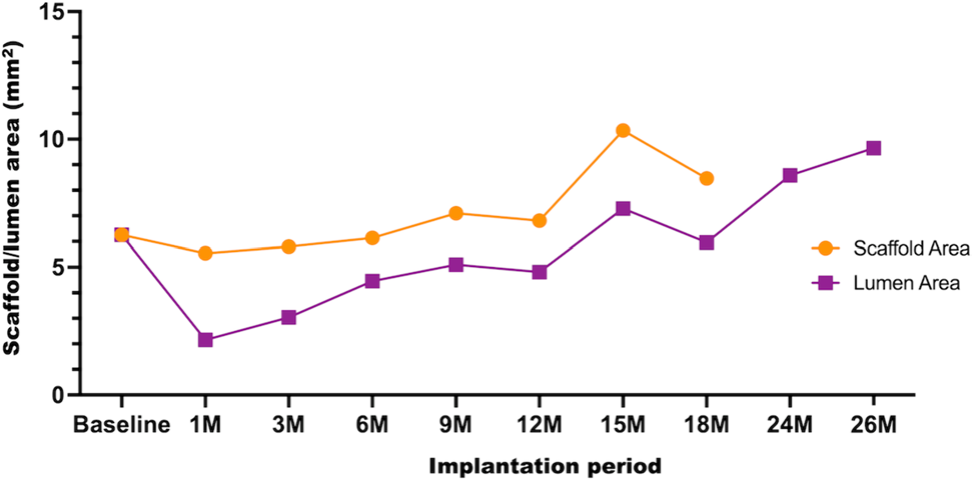
Serial OCT analysis of the JFK-PRODUCT. The data was obtained from one porcine body with two devices implanted into the left circumflex (LCX) and right coronary artery (RCA). The OCT observation was performed at every 2–3 months (M) up to 26 M. The scaffold area was not able to be measured at 24 and 26 M of implantation due to degradation of the scaffold

Acute recoil was not observed in any of the implanted scaffolds, and all animals survived the scheduled phases of the study. Angiography demonstrated thrombolysis in myocardial infarction (TIMI) 3 flow in all scaffolded vessels, with no luminal thrombus, vessel dissections, or aneurysms. The QCA analysis indicated that the median value of the diameter stenosis at 1 M and 3 M was 26.89 [interquartile range (IQR) 23.79 to 29.99] % and 19.42 (IQR: 11.48 to 29.48) %, respectively. The diameter stenosis fell by half at 12 M [8.72 (IQR: 6.87 to 9.53) %], which was followed by lumen enlargement [− 2.94 (IQR: − 5.04 to − 0.83) %] at 26 M follow-up (Table 1). A similar tendency was observed in the results of OCT analysis (Supplemental Figure S2). The late recoil at 1 M was 11.28 (IQR: 6.92 to 14.10) %, which then decreased to − 37.55 (IQR: − 46.57 to − 34.59) % at 12 M (Table 2). Serial OCT evaluation of one animal with two scaffolds in the left circumflex and right coronary arteries up to 26 M demonstrated a gradual increase in the lumen area 3 M after implantation, which peaked at 26 M, suggesting positive arterial remodeling (Fig. 1).

**Table 1:** The results of quantitative coronary angiography (QCA) after the JFK-PRODUCTS implantation

	1 M (*n* = 3)	6 M (*n* = 4)	12 M (*n* = 4)	18 M (*n* = 4)	26 M (*n* = 2)
Mean Lumen Diameter Pre (mm)	2.75 ± 0.02	2.55 ± 0.12	2.50 ± 0.03	2.68 ± 0.16	2.72 ± 0.12
Balloon to Artery Ratio	1.10 ± 0.01	1.06 ± 0.03	1.09 ± 0.01	1.05 ± 0.06	1.09 ± 0.02
Device to Artery Ratio	1.08 ± 0.01	1.06 ± 0.05	1.07 ± 0.03	1.02 ± 0.03	1.07 ± 0.01
Minimal Lumen Diameter Post (mm)	2.83 ± 0.11	2.60 ± 0.26	2.37 ± 0.12	3.02 ± 0.11	2.80 ± 0.14
Minimal Lumen Diameter at FU (mm)	1.93 ± 0.11	2.37 ± 0.14	2.37 ± 0.09	3.15 ± 0.36	2.80 ± 0.28
Diameter Stenosis at FU (%)	26.89 (23.79–29.99)	14.62 (9.77–18.02)	8.72 (6.87–9.53)	9.06 (−2.16–13.09)	−2.94 (−5.04–−0.83)
Late Loss at FU (mm)	0.97 (0.94–1.01)	0.25 (0.06–0.42)	0.00 (−0.08–0.08)	0.20 (−0.30–0.29)	0.00 (−0.05–0.05)

*FU* follow-up

**Table 2:** Results of optical coherence tomography (OCT) after the JFK-PRODUCTS implantation

	1 M (*n* = 3)	6 M (*n* = 4)	12 M (*n* = 4)	18 M (*n* = 2*)	26 M (*n* = 2)
Mean Scaffold Diameter Post (mm)	3.06 ± 0.10	2.97 ± 0.18	2.79 ± 0.11	3.02 ± 0.11	2.92 ± 0.14
Mean Scaffold Diameter Final (mm)	2.90 ± 0.08	2.84 ± 0.06	3.32 ± 0.17	NA	NA
Mean Inner Device Area Post (mm^2^)	7.40 ± 0.46	6.98 ± 0.82	6.12 ± 0.50	7.19 ± 0.53	6.72 ± 0.65
Mean Inner Device Area at FU (mm^2^)	6.61 ± 0.37	6.34 ± 0.24	8.76 ± 0.87	7.91 ± 1.74	NA
Luminal Area at FU (mm^2^)	4.40 ± 0.88	4.84 ± 0.09	6.55 ± 0.78	7.19 ± 1.41	9.16 ± 0.82
Mean Neointimal Area (mm^2^)	2.21 ± 0.53	1.50 ± 0.18	2.21 ± 0.20	1.87 ± 0.56	NA
Late Recoil (%)	11.28 (6.92–14.10)	14.43 (1.69–17.03)	−37.55 (−46.57–−34.59)	−8.47 (−30.40–13.45)	NA
Area Stenosis (%)	27.82 (27.81–36.60)	23.00 (22.53–24.43)	25.24 (24.22–26.39)	23.57 (22.37–24.77)	NA

*The data were not available in 2 vessels

**The scaffold was almost disappeared and scaffold diameter, inner device area, neointimal area, late recoil, and area stenosis was not able to measure at 26 months follow-up OCT

*FU* follow-up

## Micro-CT analysis

**Fig. 2:**
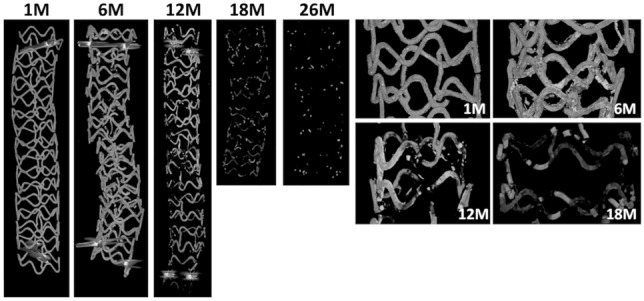
Micro-CT images of the JFK-PRODUCT in the porcine coronary arteries. The left five images are representative images for the whole scaffolds at different implantation periods. The right four images are magnified images showing the progress of the strut degradation

The representative 3D-µCT images of the JFK-PRODUCT demonstrate the progress of scaffold degradation with an increase in the implantation period (Fig. 2). At 1 M, each strut of the scaffold maintained its original width and thickness with few discontinuities. White dotty precipitates were observed on the strut surface, indicating less radiolucent substances than the Mg alloy, such as insoluble salts containing Ca and P. At 6 M, the width and thickness of the strut appeared to be wider and thicker than those at 1 M, with more uneven deposition of the less radiolucent precipitates on its surface. Some parts of the strut were observed to be darker in color, indicating its substitution with more radiolucent substances such as MgO, Mg(OH)_2_, and MgCO_3_, suggesting local degradation of the struts. At 12 M, some struts completely disappeared, while others were still in the process of degradation. Other remaining parts of the struts were completely substituted by less radiolucent substances. At 18 M, almost all crowns were dismantled and only the less radiolucent substances remained.

**Fig. 3:**
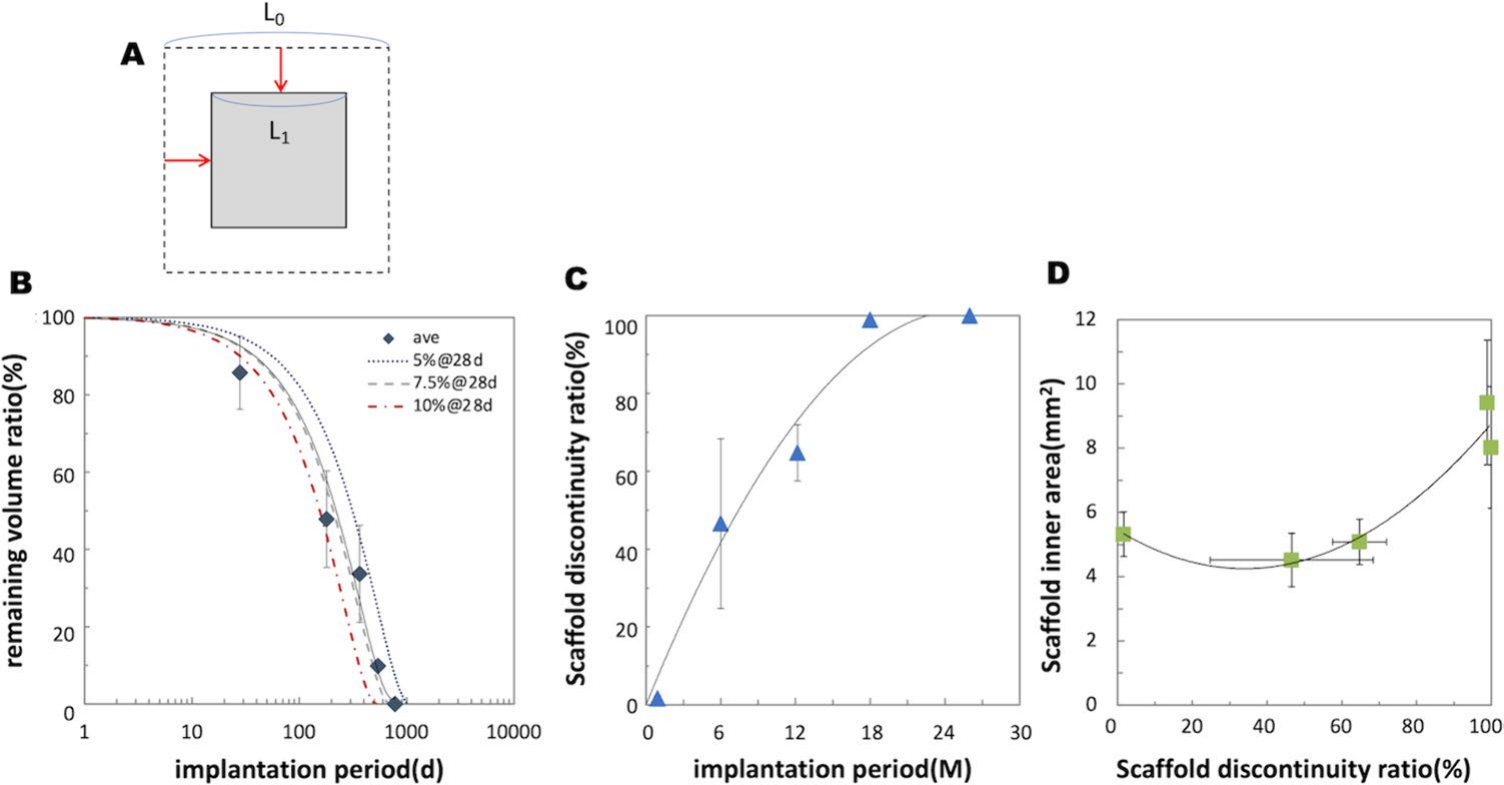
Degradation property of the JFK-PRODUCT based on the µCT images. **A** Schematic explanation of the isotropic degradation of a square-shaped strut, which is employed for the simulation of the degradation profile of a scaffold. **B** Remaining volume ratio of the JFK-PRODUCT displayed with the simulated profiles supposing a 5%, 7.5%, and 10% loss in the cross-section of the strut at 28 days of implantation. **C** The scaffold discontinuity ratio plotted against the implantation period. **D** The scaffold inner area plotted against the scaffold discontinuity ratio

Figure 3 displays the degradation profiles of the Mg alloy scaffold based on the µCT histogram analysis. As shown in Fig. 3A, we assume constant and isotropic degradation of a square-shaped strut. As the total length of the strut is much longer than its thickness and width, the reduction ratio of the strut length by degradation at both ends is negligible compared to that of the strut thickness and width. Therefore, the remaining scaffold volume can be estimated based on the cross-sectional area of the strut. Supposing the initial strut thickness and width as 0.1 mm, we can simulate the remaining volume ratio of the scaffold, as shown in Fig. 3B (details of the calculation is provided in the supplemental material). The dotted line indicates the simulation assuming a 5% decrease in the cross-sectional area of the strut at 1 M, whereas the dashed and dashed-and-dotted lines indicate a 7.5% and 10% decrease at 1 M, respectively. The actual remaining volume ratio based on the µCT images is also plotted in Fig. 3B with the approximated curve indicated as a solid line, which is consistent with the simulation of a 7.5% decrease at 1 M. This agreement indicates that the degradation behavior of the JFK-PRODUCT follows the simple hypothesis of constant and isotropic degradation. Based on this simulation, the 50% and complete degradation periods of the JFK-PRODUCT are estimated to be 7 and 23.5 M, respectively.

The scaffold discontinuity ratio is plotted against the implantation time in Fig. 3C. The scaffold discontinuity ratio was 1.6% at 1 M, which increased to 46.6% at 6 M, 64.8% at 12 M, and finally reached 97.8% at 18 M. These data indicate a loss of structural integrity at around 6–18 M, ahead of the complete resorption of the Mg alloy backbone at 26 M. Figure 3D shows a plot of the relationship between the scaffold inner area and the discontinuity ratio. When the scaffold discontinuity ratio was < 50%, which corresponded to an implantation period of 1–6 M, the scaffold inner area slightly decreased; however, when the scaffold discontinuity ratio was > 50%, which corresponded to an implantation period of > 12 M, the scaffold inner area increased even greater than that at the 1 M. This suggests that remodeling of the artery wall accompanies the loss of scaffold integrity.

## Histologic analysis

**Fig. 4:**
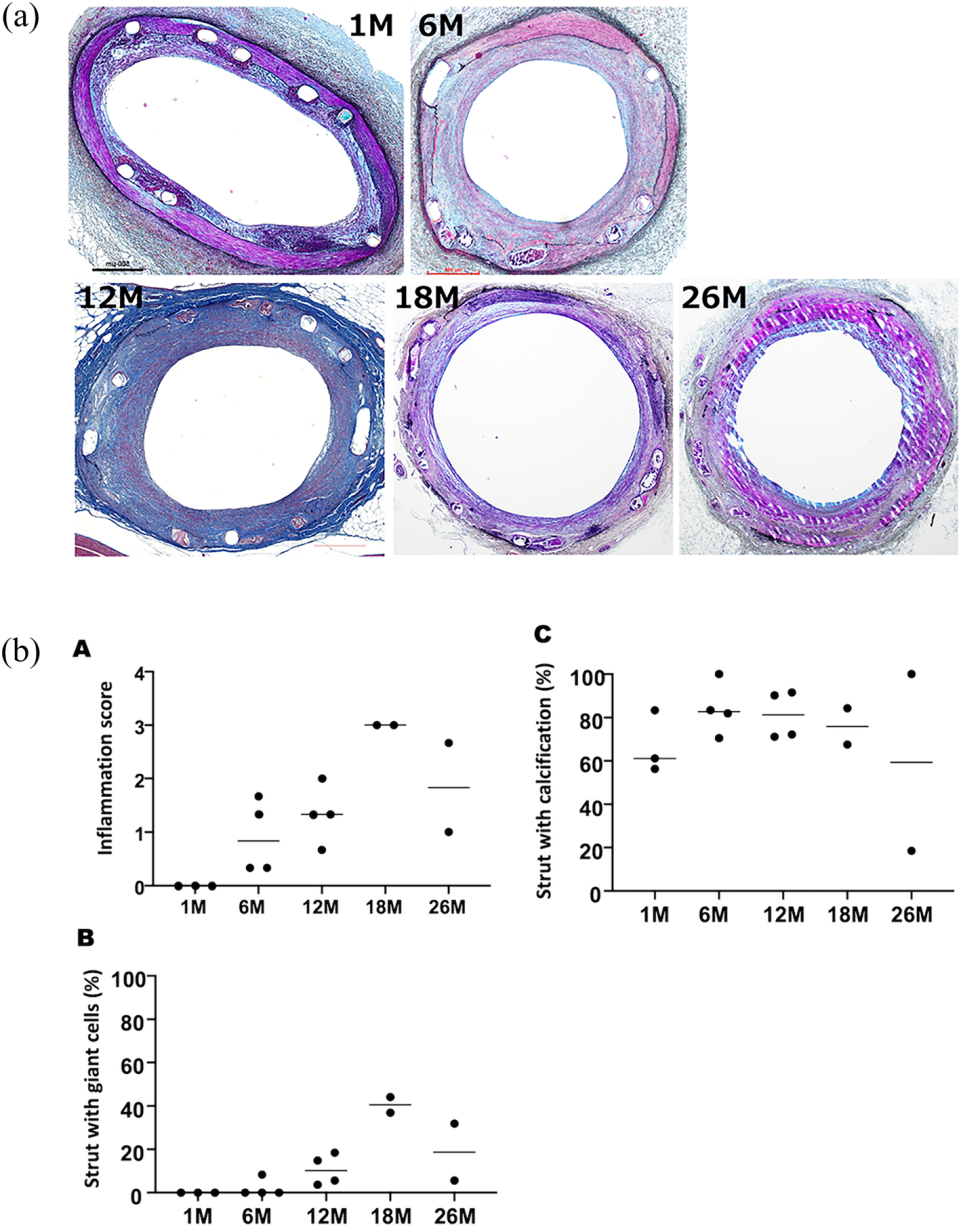
**a** Histopathological observations on the JFK-PRODUCT and vessel segments. Hematoxylin and eosin (H&E) staining images at 1, 3, 6, 12, 18, and 26 months (M) of implantation. **B** Histologic scores of the JFK-PRODUCT. **b** A: Inflammation score, B: strut with giant cells, and C: strut with calcification

All Mg alloy scaffolds were well apposed to the vessel wall, with complete endothelialization at all time points. The scaffolds were almost invisible histologically at 26 M (Fig. 4A). The inflammatory score peaked at 18 M and decreased thereafter at 26 M (Fig. 4B, Table 3). The injury score peaked at 18 M and decreased thereafter with moderate medial injury beyond 12 M. At all-time points, fibrin was rarely observed around the struts. The median value of percent struts with giant cells at 6 M was 0.0 (IQR: 0.0 to 6.2) %, which peaked at 18 M [47.1 (IQR: 38.7 to 58.9) %]. The median value of percent struts with calcification at 1 M was 61.1 [IQR: (56.3 to 83.3)] %, which peaked at 6 to 12 M [82.7 (IQR: 73.4 to 95.9) % and 81.2 (IQR: 71.4 to 91.2) %, respectively], and decreased thereafter up to 26 M.

**Table 3:** Histologic Score of the JFK-PRODUCTS

		1 M (*n* = 3)	6 M (*n* = 4)	12 M (*n* = 4)	18 M (*n* = 4)	26 M (*n* = 2)	*p*
Morphometric analysis	External elastic lamina area (mm^2^)	6.9 (6.0–9.5)	8.6 (6.5–13.7)	6.3 (1.8–7.6)	8.3 (5.0–11.6)	7.5 (6.5–8.4)	0.3
	Scaffold area (mm^2^)	5.9 (4.7–6.8)	7.3 (4.9–11.5)	5.6 (5.0–6.7)	7.6 (4.1–10.2)	6.1 (5.1–7.1)	0.4
	Lumen area (mm^2^)	3.9 (2.8–4.6)	4.3 (2.4–6.9)	3.3 (2.5–4.9)	4.8 (1.7–6.9)	3.1 (2.8–3.4)	0.4
	Underlying plaque area (mm^2^)	2.0 (1.9–2.2)	2.6 (2.0–5.6)	2.3 (1.8–2.4)	2.8 (2.3–3.3)	3.0 (2.3–3.7)	0.2
	Media area (mm^2^)	1.3 (1.1–2.7)	1.7 (1.1–2.2)	0.9 (0.7–0.9)	1.0 (0.6–1.4)	1.4 (1.4–1.4)	0.1
	Maximum percent stenosis (%)	34.6 (32.9–40.9)	51.7 (28.0–54.3)	41.3 (28.6–50.1)	36.8 (32.6–62.0)	48.6 (45.3–51.9)	0.4
	Mean neointimal thickness (mm)	0.3 (0.2–0.4)	0.3 (0.2–0.5)	0.2 (0.2–0.3)	0.3 (0.3–0.3)	0.4 (0.3–0.5)	0.4
Pathologic analysis	Injury Score	1.6 (1.5–1.8)	1.9 (1.8–2.0)	2.2 (2.0–2.3)	2.3 (1.8–2.5)	2.2 (2.0–2.5)	0.02
	Strut with Fibrin (%)	0.0 (0.0–4.2)	0.0 (0.0–0.0)	0.0 (0.0–0.0)	4.1 (0.0–13.9)	0.0 (0.0–0.0)	0.4
	Fibrin score	0.0 (0.0–0.0)	0.0 (0.0–0.0)	0.0 (0.0–0.0)	0.2 (0.0–0.6)	0.0 (0.0–0.0)	1.0
	Inflammation score	0.0 (0.0–0.0)	0.8 (0.3–1.6)	1.3 (0.8–1.8)	2.7 (2.1–3.0)	1.8 (1.0–2.7)	0.03
	Strut with inflammatory cells (%)	0.0 (0.0–0.0)	17.8 (3.7–31.2)	24.6 (10.4–38.2)	66.4 (52.7–88.5)	35.9 (17.8–54.1)	0.04
	Strut with giant cells (%)	0.0 (0.0–0.0)	0.0 (0.0–6.2)	10.2 (4.2–17.6)	47.1 (38.7–58.9)	18.7 (5.6–31.8)	0.03
	Strut with calcification (%)	61.1 (56.3–83.3)	82.7 (73.4–95.9)	81.2 (71.4–91.2)	70.5 (26.0–81.5)	59.3 (18.5–100.0)	0.6

## SEM–EDX analysis

**Fig. 5:**
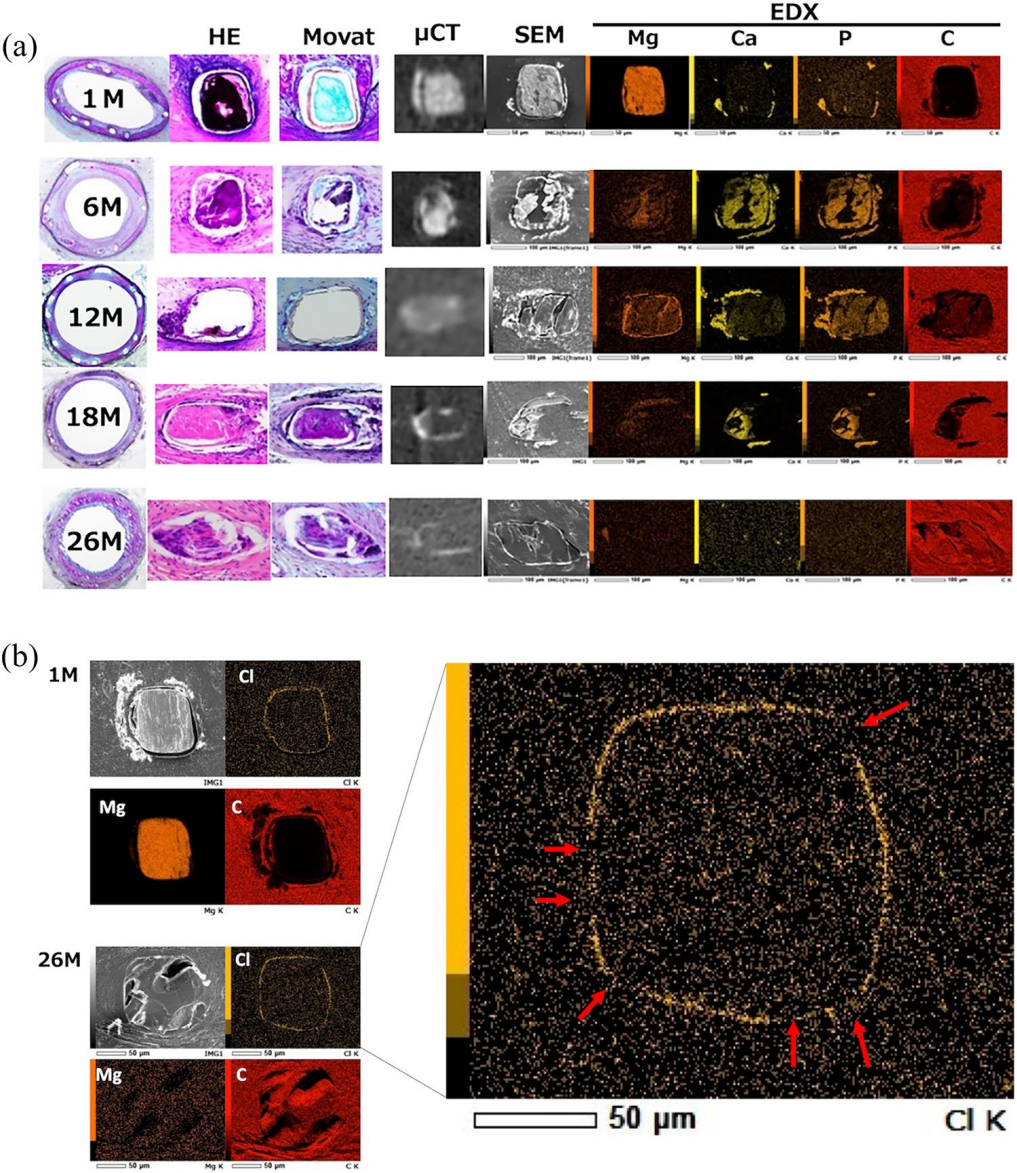
**A** Co-registration of the representative images of histology, OCT, µCT, and SEM–EDX at different implantation periods. **B**. SEM–EDX images of the strut with distributions of Cl, Mg, and C. The red arrows on the magnified images of the Cl distribution at 26 M indicate the disruption of the Parylene C layer on the strut surface

SEM–EDX evaluation of the struts showed that the detected elements varied with the period of implantation (Fig. 5A). At 1 M, only Mg was detected in the strut center, whereas Ca and P were observed in the strut periphery. At 6 M, the struts partially disappeared, and Ca and P were observed in the strut region, indicating the degradation of the Mg alloy backbone. At 18 M, the proportion of carbon (C) in the strut region further increased, whereas those of Ca and P decreased with less detection of Mg. At 26 M, most of the strut region was replaced by C derived from the vascular tissue. These observations indicate a synchronous transition of Ca and P in the strut region, suggesting the formation of calcium phosphate associated with Mg alloy corrosion and its resorption in the later phase of degradation. Further observation of the strut regions at 26 M revealed a partial loss of Cl, which constitutes Parylene C (Fig. 5B), indicating the degradation and disappearance of the Parylene C coating in porcine artery tissue. These findings support the complete resorption of the JFK-PRODUCT, including calcium phosphate and the coating layers on its strut surface.

## Discussion

In the current study, we performed a comprehensive analysis of the JFK-PRODUCT using angiography, OCT, µCT, histopathology, and SEM–EDX analysis in healthy porcine coronary arteries at multiple time points ranging from 1 to 26 M. The detailed characteristics of the JFK-PRODUCT have been reported previously [13, 14]. The JFK-PRODUCT uses thin struts (110 µm) and a RE-free Mg alloy with fluorine treatment followed by a Parylene C coating, which allows the scaffold to be completely resorbed in ~ 2 years, with no remaining calcium phosphate in the tissue. The principal findings of this study can be summarized as follows: 1) angiography and OCT analysis confirmed vascular patency without acute recoil throughout the study in all the scaffolds implanted; 2) vessel enlargement was observed at 6 M, and this trend was further augmented beyond 12 M; 3) µCT, SEM–EDX, and histopathological analysis confirmed partial substitution of the Mg alloy strut by insoluble substances containing Ca and P at 6 M as an initial phase of degradation, while further degradation with strut discontinuity was observed at 12 M, followed by complete resorption at 26 M; and 4) the inflammatory response increased from 6 M, peaked at 18 M, and decreased thereafter.

The complete resorption of the scaffold, including calcium phosphate, was confirmed in the current animal study. As shown in the µCT images and SEM–EDX analysis, deposition of Ca and P was observed at 1 M, mainly outside the coating layer. Substitution of the Mg alloy backbone by calcium phosphates partially occurred at 6 M and longer implantation periods, which is a similar process to the currently available Mg alloy BRS (Magmaris) [10]. The calcium phosphate deposition of the JFK-PRODUCT peaked at 6–12 M and disappeared at 26 M. Histological evaluation, however, shows calcification in 59.3% of struts at 26 months (Table 3), which may represent a transitional state from calcium to carbon. In contrast, calcium phosphate deposition associated with the Magmaris scaffold remained even 2 years after implantation in micro CT analysis [10]. This can be attributed to the difference in Mg alloy backbones, in that the JFK-PRODUCT employs an original RE-free alloy (Mg-1.45%Zn-0.14%Mn) [14], whereas the Magmaris employs an alloy with RE elements such as Y (4 wt%), Dy (0.5 wt%), Nd (2 wt%), and Gd (0.5 wt%) [22]. The DREAMS 3G, the latest version of the Magmaris-BRS, employs an RE-free, Mg–6.25% Al alloy [12], but a recent preclinical study demonstrated its complete substitution by calcium phosphate (containing Al) at 12 M after implantation into porcine coronary arteries [12]. The JFK-PRODUCT is a unique Mg alloy BRS that achieves almost complete resorption, leaving no calcium phosphate at 26 M after implantation.

In the QCA results of the JFK-PRODUCT, the diameter stenosis was approximately 25% at 1 and 3 M, and it was significantly reduced to approximately one third at the 12 M follow-up. Comparing these results to those of a previous Magmaris preclinical study [23], the findings favor the Magmaris at the beginning of the implantation period, before reversing after 12 M. This may be attributed to the influence of the polymer coating on the BRSs at the early stages of implantation, whereas at the later stage, the degradation of the polymer itself and other factors, such as moderate medial damage due to moderate inflammation secondary to the degradation process, may contribute to the improvement in stenosis and vessel enlargement. This trend was also observed in the recoil rate by OCT analysis. This tendency is consistent with the time course of negative and positive vascular remodeling, which is an inherent vascular behavior in porcine coronary models, as revealed in previous studies [24–26]. Further studies in the clinical settings are needed to evaluate the relationship between vessel enlargement and degradation of the polymer coating.

An overview of the degradation process of the JFK-PRODUCT is illustrated in Graphical Abstract, with serial changes observed in the scaffold/lumen area obtained by OCT, the remaining scaffold volume ratio, and the scaffold discontinuity ratio obtained by µCT. The results revealed an initial reduction in the scaffold/luminal area at 3–6 M, followed by a secondary increase at 18 and 26 M, indicating positive remodeling due to vessel uncaging by the loss of scaffold integrity. This enlargement timing was slightly slower than that of the Magmaris (12 M) [23], which may be attributed to the slower degradation of the JFK-PRODUCT than the Magmaris. The slower controlled degradation of the JFK-PRODUCT may be due to the surface treatments with fluorination and Parylene C coating, which improved the corrosion resistance of the Mg alloy scaffold by in vitro testing [14]. Parylene C is a commonly used surface coating material for medical devices such as stents and pacemakers, with no previously reported hazards and toxicity.

## Study limitations

The current study has several limitations that warrant discussion. First, the number of scaffolds evaluated was relatively small. Second, this study was conducted using a healthy porcine coronary artery model; therefore, the results cannot consider the effect of atherosclerosis on the degradation of and tissue response to the JFK-PRODUCT. Nevertheless, serial follow-up with detailed evaluation using several imaging modalities provides mechanistic and biological insight into the degradation process of the JFK-PRODUCT.

## Conclusions

In the current preclinical study using a healthy porcine coronary artery model, a novel Mg alloy-based BRS, JFK-PRODUCT, demonstrated its safety with complete resorption of the Mg alloy backbone, leaving no calcium phosphates in the tissue at 26 M. Further clinical studies are needed to confirm the findings of this preclinical study.

## Supplementary Information

Below is the link to the electronic supplementary material.

Supplementary file1 (DOCX 7091 KB)

## Data Availability

The data that support the findings of this study are available from the corresponding author upon reasonable request. Due to the sensitive nature of the clinical data, it has not been made publicly available. Researchers who wish to access the data should contact Gaku Nakazawa at [gnakazawa@med.kindai.ac.jp] to discuss the conditions under which the data can be accessed.

## Declarations

### Generative AI and AI-assisted technologies in the writing process

Generative AI and AI-assisted technologies were not used in the current manuscript.
