# Supplementary material for: Degradation of a novel magnesium alloy-based bioresorbable coronary scaffold in a swine coronary artery model
Source: Cardiovasc Interv Ther. 2024 Jul 22;39(4):428–37. doi: 10.1007/s12928-024-01023-3 (PMC11436393; doi:10.1007/s12928-024-01023-3)
Supplement: Supplementary file 1 — Supplementary file1 (DOCX 7091 KB) [file 12928_2024_1023_MOESM1_ESM.docx]

**Supplemental materials**

Detailed methods with 2 supplemental figures and 1 supplemental table

**Supplemental Figure S1**. **Details of the JFK-PRODUCT**

The JFK-PRODUCT employs a magnesium-based alloy that is free of rare earth elements such as yttrium (Y), dysprosium (Dy), neodium (Nd), and gadolinium (Gd). Base alloy has a precise fluoride and Parylene C treatment to improve corrosion resistance. The outer layer is further coated with Sirolimus, together with a polymer layer combining poly (DL-lactide-co-ε-caprolactone) and poly (DL-lactide) or poly (ε-caprolactone) on top of it to improve its corrosion resistance and anti-inflammatory properties. (A) The scaffold pattern of an open cell with a six-crown two-link. (B) The details of the strut structure.

**Detailed methods**

**Experimental model**

The study protocol was reviewed and approved by the Institutional Animal Care and Use Committee at AccelLAB (Quebec, Canada) and Gateway Medical Innovation Center (Shanghai, China). A total of 14 porcine received the JFK-PRODUCTS in a total of 17 coronary arteries, and sacrificed at 28 days [abbreviated as 1 month (M), n=3], 180 days (6 M, n=4), 365 days (12 M, n=4), 540 days (18 M, n=4), and 780 days (26 M, n=2) after the scaffold implantation (Supplemental Table 1). For each procedure, anesthesia induction for tracheal intubation was achieved with propofol injected intravenously (IV). Upon induction of anesthesia, the porcine was intubated and supported with mechanical ventilation. Isoflurane in oxygen was administered to maintain a surgical plane of anesthesia. In order to prevent pain sensitization and minimize postoperative pain, buprenorphine HCl was administered intramuscularly (IM) as preemptive analgesia for implantation. Open carotid artery access was secured, and heparin (100–300 IU/kg) was administered intravenously and activated clotting time (ACT) was measured at least every 30 minutes and recorded. In case of ACT shorter than 300 seconds, additional heparin was administered.

**Supplemental Table 1**

| duration | animal no. | Coronary arteries | | |
| --- | --- | --- | --- | --- |
|  |  | RCA | LAD | LCX |
| 1M | animal 1 |  |  | JFK-PRODUCT |
|  | animal 2 |  |  | JFK-PRODUCT |
|  | animal 3 |  |  | JFK-PRODUCT |
| 6M | animal 4 |  |  | JFK-PRODUCT |
|  | animal 5 | JFK-PRODUCT |  |  |
|  | animal 6 |  | JFK-PRODUCT | JFK-PRODUCT |
| 12M | animal 7 |  |  | JFK-PRODUCT |
|  | animal 8 |  | JFK-PRODUCT |  |
|  | animal 9 | JFK-PRODUCT |  |  |
|  | animal 10 |  | JFK-PRODUCT |  |
| 18M | animal 11 | JFK-PRODUCT |  | JFK-PRODUCT |
|  | animal 12 |  |  | JFK-PRODUCT |
|  | animal 13 |  | JFK-PRODUCT |  |
| 26M | animal 14 | JFK-PRODUCT |  | JFK-PRODUCT |

**Angioplasty**

Under fluoroscopic guidance, a guide catheter (6 Fr) was inserted through the sheath and advanced to the appropriate location. After placement of the guide catheter, nitroglycerin was delivered intracoronary (IC) to achieve coronary vasodilatation. Angiographic images of the vessel were obtained with contrast media to identify the proper location for the deployment site (designated pre-treatment angiographies). A guidewire was inserted into the chosen artery. QCA was performed at this time to document the reference diameter for device placement.

After pre-treatment angiography was obtained, the scaffold was deployed according to the compliance charts as a guide to achieve a balloon to artery ratio from 1.05 to 1.10:1 based on QCA measurement, and the pressure was maintained for at least 30 seconds.

**Scaffold harvest**

The scaffolds were harvested at the designated time point. After nitroglycerin was delivered to the treated arteries, angiography and OCT evaluation was performed. Euthanasia (IV overdose of barbiturates) was performed by administering a lethal injection of pentobarbital (~110 mg/kg) as a rapid bolus. Hearts were harvested and perfused with lactated Ringer’s solution, then fixation perfusion was performed with neutral buffered formalin (NBF). Treated vessels were exposed to NBF for approximately 24 ± 4 h before further processing.

**Quantitative coronary angiography (QCA)** **analysis**

QCA were performed using Medis QAngio® XA 7.3 system (Medias, Leiden, the Netherlands) as previously reported. For angiograms recorded at orthogonal angles in the coronary arteries, the mean values of parameters measured or calculated from the 2 angles were reported. Various parameters such as mean lumen diameter, minimal lumen diameter (MLD), maximal lumen diameter of the target region, the reference vessel diameter (RVD), device to artery ratio were measured. Diameter stenosis was decided as [1 − (MLD/RVD)] × 100 (%), where RVD was the reference diameter at the position of the obstruction. Late lumen loss was calculated by subtracting MLD final from MLD post-treatment.

**Optical coherence tomography (OCT)** **analysis**

OCT imaging was performed with the ILUMIEN or ILUMIEN Optics imaging system (Abbott Cardiovascular, Plymouth, USA). After the scaffold implantation, OCT analysis was performed for all treated vessels. Vessel patency, scaffold and strut adherence to vessel wall and constructive change of the scaffold struts were evaluated based on OCT images of implanted vessel sections. OCT was also performed before euthanasia.

Scaffold diameter, inner device area, and luminal area were calculated by the average of the data measured on 5 slices in the scaffolded segment with an interval of 2–3 mm at the post-treatment or final OCT observation. Neointimal area was calculated by the average of the differences between the mean inner device area and luminal area on the selected 5 slices in the scaffolded segment with an interval of 2–3 mm at the final OCT observation. Late recoil was calculated by the average of the values calculated by the following equation; {[1 - (Mean Inner Device Area Final /Mean Inner Device Area Post-Treatment)] × 100} for 5 selected slices in the scaffolded segment with an interval of 2–3 mm at the post-treatment or final OCT observation. Area stenosis was calculated by the average of the values calculated by the following equation; [(Mean Inner Device Area Final/Luminal Area Final) ×100] for 5 selected slices in the scaffolded segment with an interval of 2–3 mm at the final OCT observation.

**Micro-focus X-ray computer tomography (µCT) analysis**

Before histological processing, the degradation of the scaffold and strut discontinuity were analyzed based on µCT observation (SMX-90CT, Shimadzu, Kyoto, Japan, or XT H225ST, Nikon, Tokyo, Japan) at 1, 6, 12, 18, and 26 M after implantation (n=3, 4, 4, 2 and 2, respectively). Arterial segment treated with a scaffold was underwent a full scan at an optimal condition for each of SMX-90CT or XT-H225ST system with the resolution of 4.4–6.4 or 11.6‒15.0 µm/voxel, respectively. The remaining volume of the scaffold was estimated based on histogram data using a peak fitting program (4). The area of corresponding peak was converted into the volume by multiplying with the voxel volume and then, standardized by that of an intact scaffold. Strut discontinuity was manually analyzed on the 3D images visualized by an image analysis software (VGStudio MAX3.0, Volume Graphics, Heidelberg, Germany) as a fracture line on the strut or displacement of the strut fragment. A number of half-crowns or links without strut discontinuity, *N*_nd_ was counted to give the scaffold discontinuity ratio of the whole scaffold, *F* by the following equation;

*F*(%) = [1 − (*N*_nd_/ *N*_total_)] × 100

where *N*_total_ indicates the total number of half-crowns and links in the scaffold.

The scaffold inner area was measured on the 2D sectional images prepared at the approximately 3 mm intervals from the proximal end of the scaffold. Five sections were analyzed per one scaffold sample.

**Histological preparation and assessment**

For light microscopy, the treated vessel segments were dehydrated in a graded series of ethanol and embedded in Spur’s epoxy resin. After polymerization, proximal, middle and distal segments, approximately 2 to 3 mm, were sawed from the devices. Sections from the implants were sliced by a rotary microtome at 4 to 6 µm, mounted and stained with Hematoxylin and Eosin (H&E), Elastin Trichrome (ET), and modified Movat Pentachrome stains.

Morphometric analysis and histologic assessment of scaffolded sections were performed as previously described (1-3). In brief, morphometric measurements were performed with an image-analysis software (Zen2, Zeiss, Oberkochen, Germany). Measurements included the cross-sectional areas cut from the scaffolded vessel (proximal, mid, and distal) for external elastic lamina, scaffold, and lumen areas. Neointimal thickness was measured and defined as the distance from the abluminal surface of each strut to the luminal border. Mean neointimal thickness was the average of measurements taken at each strut. Uncovered struts were identified by the presence of platelet and/or fibrin thrombus or bare struts with absence of neointima, and the ratio of uncovered-to-total scaffold struts per section was calculated and expressed as percent.

Histologic assessment such as vessel injury and inflammation score were semi quantitatively scored for each section. Struts with fibrin, >10 inflammatory cells, giant cells, and calcification were counted and expressed as percent as previously described (1-3).

**Detailed in simulation of scaffold volume along its degradation**

As shown in Figure 5A in the main text, we suppose a constant and isotropic degradation of a square-shaped strut to simulate the scaffold volume during the degradation process. Since the total length of the strut is much longer than its thickness and width, the reduction ratio of the strut length by the degradation at the both ends are negligible comparing to those of the strut thickness and width. Therefore, a remaining scaffold volume can be estimated based on the cross-section area of the strut as follows;

*S* = *L_1_*^2^ = (*L_0_* − 2*Vt*)^2^

where *S* represents the cross-section area (mm^2^); *L_0_* and *L_1_* indicate the strut thickness (= width) at an initial and a certain time point (mm), respectively; *V* means degradation rate (mm/d); *t* indicates degradation period (d). Supposing *L_0_* as 0.1 mm, we can simulate the remaining volume ratio of the scaffold as shown in the Figure 5B in the main text.


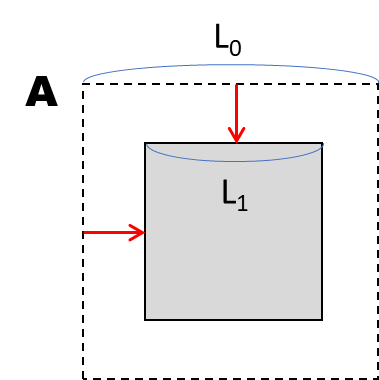

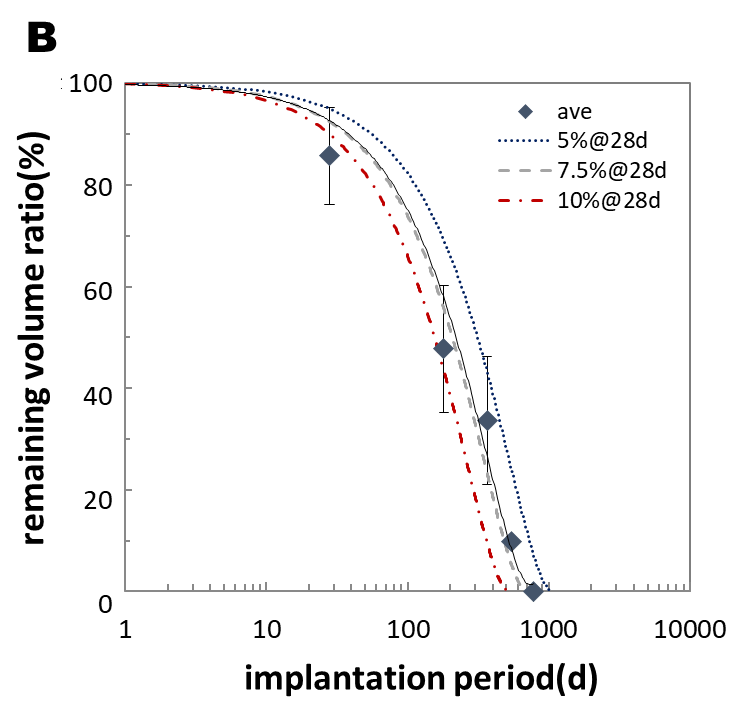


Figure 5 AB (redisplayed). Degradation property of JFK-PRODUCTS based on the µCT images. A) Schematic explanation of the isotropic degradation of a square-shaped strut, which is employed for the simulation of the degradation profile of a scaffold. B) Remaining volume ratio of the JFK-PRODUCTS displayed with the simulated profiles supposing 5%, 7.5%, and 10% loss in the cross-section of the strut at 28d of implantation.

**Supplemental Figure S2. Representative serial OCT images of the JFK-PRODUCT**

The test device was implanted into the porcine coronary arteries up to 26 months.

**References**

1. Nakazawa G, Shinke T, Ijichi T et al. Comparison of vascular response between durable and biodegradable polymer-based drug-eluting stents in a porcine coronary artery model. EuroIntervention 2014;10:717-23.

2. Torii S, Yahagi K, Mori H et al. Biologic Drug Effect and Particulate Embolization of Drug-Eluting Stents versus Drug-Coated Balloons in Healthy Swine Femoropopliteal Arteries. J Vasc Interv Radiol 2018;29:1041-1049.e3.

3. Torii S, Yahagi K, Mori H et al. Safety of Zilver PTX Drug-Eluting Stent Implantation Following Drug-Coated Balloon Dilation in a Healthy Swine Model. J Endovasc Ther 2018;25:118-126.

1. Wojdyr M. Fityk : a general-purpose peak fitting program. *Journal of Applied Crystallography*. 2010 2010;43:1126-1128.
